# Behavior and Health Indicators to Assess Cull Cow's Welfare in Livestock Markets

**DOI:** 10.3389/fvets.2020.00471

**Published:** 2020-08-07

**Authors:** Melissa Sánchez-Hidalgo, Viviana Bravo, Carmen Gallo

**Affiliations:** ^1^Instituto de Ciencia Animal, Facultad de Ciencias Veterinarias, OIE Collaborating Centre for Animal Welfare and Livestock Production Systems—Chile, Universidad Austral de Chile, Valdivia, Chile; ^2^Escuela de Graduados, Facultad de Ciencias Veterinarias, Universidad Austral de Chile, Valdivia, Chile

**Keywords:** cull cows, livestock market, welfare indicators, behavior, handling, health

## Abstract

The welfare status of cull cows going through livestock markets was assessed in 12 premises in Chile, using behavioral and health indicators observed during unloading, auction, and loading (once in winter and once in summer). Groups of cows were observed by the same observer and the following indicators were recorded at each stage: slips, falls, balks, turns, jumps, and vocalizations of cows were considered as behavioral events and a proportion was calculated based on the number of observed events per group divided by the number of cows per group to give a behavioral event index (BEI). Health status of the cows was assessed during auction by recording the proportion of cows with low body condition, lameness, udder problems, tegumentary lesions, and tail abnormalities. Handler behavior was assessed using a count of negative tactile interactions (NTI) with the cows, like blows/hits, kicks, and pokes with the devices used to drive them, then a NTI index (NTII) was calculated as for BEI. Using the groups of cows as the statistical unit, statistical models were built and used to identify how NTII, some facilities features and comingling were associated with BEI registered during unloading, auction, and loading and also to calculate if the selling price was associated with the different health problems of cows, using the software MLwiN 3.03. A total of 1,103 groups of cows (*n* = 3,963 cows) were observed, finding a high percentage of slips and balks, whereas hitting and poking were frequent NTI. The highest mean BEI was observed during unloading in winter (1.10), whilst the lowest one was found during auction in summer (0.34). There was an increase of 0.11 in BEI for every extra unit increase in the NTII by the handlers. The BEI was negatively affected by the winter season compared to summer. Of 1,608 cows, 49.8% had a low body condition, 28.3% had udder problems, 24% were lame, 8.7% presented tegumentary lesions, and 3.1% tail abnormalities. It can be concluded that the health of the cull cows is already compromised when leaving the farms; cow behavior and handler tactile interactions with the cows are useful indicators to assess the welfare of cull cows at livestock markets.

## Introduction

Livestock markets are a traditional scenario to sell and buy cattle, and in Latin American countries are an important way for small farmers to sell their products (1). However, compared to selling directly from farm to farm or from farm to slaughterhouse, selling through auction markets implies greater concerns in terms of risks of transmission of diseases (2), increase in carcass bruising (3), and possibly greater animal welfare problems, considering that handling events during loading, transport, and unloading are at least duplicated and can generate high levels of stress (4).

Gallo and Tadich (5) indicate that comingling animals from diverse origins as it occurs at livestock markets implies an additional physiological and behavioral stress for the animals that can cause fatigue, fear, dehydration, hunger, weight loss, and lesions. It is well-known that the prevalence of bruises in carcasses of cattle that have undergone auction commercialization is higher than in carcasses of cattle sold directly from farms to slaughterhouses (3, 6, 7). The increased prevalence of bruises can be attributable to the fact that cattle sold through livestock markets undergo more handling events and have more human-animal, animal-animal, and also animal-facilities interactions (3, 8).

In Canada, sick dairy cows are an important welfare problem identified at livestock markets, particularly when the milk quota was reduced (4). In the United Kingdom the main welfare problem of livestock markets has been related to handling (6) and to infrastructure (9). In Chile De Vries (10) and Sepúlveda (8) found that in general, inadequate infrastructure and lack of trained personnel were relevant features at markets. Studies in livestock markets in the central region of Mexico (1, 2), have shown that animals present severe physiological, metabolic, and behavioral changes. In a recent study Bravo et al. (11) registered the behavior of weaned calves sold through livestock markets, as observed at the stages of unloading, grading, auction, loading, and penning and found that the main factors associated with poor welfare of the calves, according to their behavioral indicators, were related to bad handling techniques, infrastructure deficiencies, and lack of training of personnel.

Adult and old cows that are culled from beef and dairy farms represent between 30 and 40% of all animals sold through livestock markets in Chile (12). The physical and physiological conditions of culled cows differ widely from the younger and healthier cattle categories (13). Moreover, due to their comparatively lower commercial value, culled cows are usually handled with less care, are kept longer in lairage pens (14, 15), and show more bruises on their carcasses compared to steers and heifers (16, 17). At arrival at the slaughterhouse, the presence of health problems, a low body condition, and udder problems of culled cows also act as risk factors for the severity of bruises and carcass condemnations (18), increasing economic losses in the meat industry.

Although the effects of livestock markets on meat quality after slaughter are well-known, there is no sufficient scientific evidence regarding the effects of marketing on direct welfare indicators of the cows while alive. Therefore, the objective of the present study was to evaluate the welfare status of cows going through livestock markets in Chile, using behavioral, health, handler, and facilities indicators observed during the unloading, auction and loading process.

## Materials and Methods

The methodology used was similar to that described by Bravo et al. (11) in weaned calves and the evaluation guidelines were developed based on the recommendations of Welfare Quality (19) and Grandin (20) for slaughter plants, the current Chilean Animal Protection Law (21) and pilot visits to markets. Twelve auction markets in the southern regions of Chile (Geographical coordinates: 37.81208°S−72.67112°W to −45.61736°S−72.10496°W) were selected after pilot visits to the 21 existing southern markets; this selection was based on availability (auctioning weekly all year round or at least fortnightly) and on the number of cows arriving during 1 auction day (>50 cows). In order to evaluate possible seasonal differences, the markets were visited twice, the first visit was performed in summer and the second in winter. This considered mean daily temperatures (13.5 vs. 5.9°C) and pluviosity (31.6 vs. 194.7 mm) during summer (Nov-Dec) and winter months (June-July), respectively. The criterion to define cull cows at arrival at the market was a female bovine with some development of the udder, showing that she had had at least one calving, sometimes even accompanied by the calf.

The passage of the animals through the market was assessed in three stages, which are defined in Table 1. In each stage, groups of cows were observed by a veterinarian, trained in animal behavior, and welfare, in order to measure behavioral indicators of welfare during unloading, auction, and loading. Some other features related with facilities and handling by the personnel were also assessed independently in each group of cows observed at each market and stage; thus, animals were handled under comparable but not identical conditions at the different markets. To maintain consistency, the distinct stages were always assessed by the same observer throughout the study.

**Table 1:** Definition of the stages evaluated in livestock markets.

**Stage**	**Description**
Unloading	From the moment truck doors are opened until the last animal comes off the vehicle.
Auction	From cows entering the auction ring until leaving it.
Loading	From the lead animal moving toward the truck until the last animal is loaded into the truck.

To assess the cow's welfare in each stage, the following indicators were evaluated.

### Behavioral Indicators of the Cows

These were quantified in each observed group of cows, at each stage, in accordance with definitions provided by Maria et al. (22) and Gregory et al. (9), by counting slips, falls, balks, turns, jumps, and vocalizations. The number of individuals per group was also recorded. This included a count of other species involved during unloading as necessary, as on occasion, mixed species groups could arrive at the market.

#### Slips

Foot slide or stumbling that did not result in a fall but nearly did so.

#### Falls

The cow went down on its side or both knees, or was off both its hind feet.

#### Balks

An unwanted arrested flow due to an apparent distraction or intimidation.

#### Turns

When the animal changes direction of movement against the animal flow.

#### Jumps

Leaping with all four feet simultaneously off the ground in a manner or situation that could be hazardous for the cow.

#### Vocalizations

When a cow makes a vocal sound (mooes).

### Health Indicators of the Cows

The following health indicators were registered during auction, in accordance with definitions provided by Sánchez-Hidalgo et al. (18).

#### Body Condition

according to the European Welfare Quality (2009) protocol (19), a value of 0 was assigned to cows with a normal (regular) body condition, one to very lean cows (indicators for “very lean” present in at least three body regions) and two to very fat cows (indicators for “very fat” present in at least three body regions).

#### Abnormality of Tail

This was registered when the cows presented visually an amputated (shortened) or fractured tail (noticeable as an increase in volume and/or a lack of smooth continuity along the vertebrae when the tail was hanging relaxed). Fractured tails are common feature as a result of twisting tails by inappropriate handling.

#### Tegumentary Lesions

They were registered as such when the cows presented areas of alopecia in tarsus, hindquarters, carps, neck, shoulders, and back, as well as abrasions, scars, lacerations, hyperkeratosis, and other type of open wounds on their body.

#### Lameness

Category 0 without lameness was assigned to cows when timing of steps and weight-bearing was equal on all four feet. And one to cows with lameness (including categories 1 and 2 of the Welfare Quality Protocol, 2009) [i.e., if there was imperfect temporal rhythm in stride creating a limp or strong reluctance to bear weight on one limb, (19)].

#### Udder Problems

Cows presenting an increased volume of one or several mammary gland quarters, increased redness and inflammation of some quarters suggestive of mastitis, damaged, or visibly dry quarters, teats with visible wounds.

Additionally, during auction the price per kg live weight of the culled cows was registered in order to analyze the relationship between health indicators and price of the cows.

### Handler Indicators

Handler-behavior was measured using a count of negative tactile interactions with the cows (blows/hits, kicks, and pokes with the devices used to drive the animals) during each stage, in accordance with definitions provided by Strappini et al. (3).

#### Blows/Hits

A person hits the animal with a hard object (usually wooden sticks).

#### Kicks

A person beats the animal with a foot.

#### Pokes

A person sticks a pointed object in the animal's body (usually pointed wooden sticks).

### Additional Features

The facilities and comingling were also evaluated as possible features that could affect and/or disrupt the normal passage of the animals being driven.

#### Facilities

Floor type (slip-proof/slippery), obstacles/distractors in the path of the cows (presence or absence), and the slope of the ramp used (yes/no) (23) were recorded for each stage per group. Ramp slopes were obtained by simply measuring the height and the length of the ramp using a tape measure.

#### Comingling

In each stage it was recorded if cows were mixed with other categories of cattle, (e.g., heifers, bulls, steers, and calves) or with other animal species, (e.g., pigs, sheep, and horse) (yes/no).

### Data Analysis

The slips, falls, balks, turns, and vocalizations were considered as behavioral events (BE). For each type of event, a proportion was calculated based on the number of observed events per group divided by the number of cows per group to give a behavioral event index (BEI). The hits, kicks and pokes by handlers with the device used to drive the animals, were classified as negative tactile interactions (NTI) and for each type of interaction, a proportion was calculated based on the number of observed interactions per group divided by the number of cows per group to give a NTI index (NTII). A descriptive analysis (mean, standard deviation (*SD*), minimum and maximum values and percentage calculation) was performed using IBM SPSS Statistics version 25, The body condition, abnormalities of tail, tegumentary lesions, lameness, and udder problems observed during the auction stage were counted as health indicators; one cow could have more than one health problem.

Statistical models were built and used to identify how NTII, some facilities features and comingling were associated with BEI as registered during unloading, auction, and loading. And also to calculate if the selling price was associated with the different health problems of cows. Multilevel model analyses were performed using the software MLwiN 3.03 (24). A multilevel modeling approach was employed to account for the clustering as a random effect and the repeated measurement structure of the data, (e.g., groups within auction market and repeated visits to market). Predictor variables were retained within models at α ≤ 0.05. A graphical inspection of the residuals was made to check for normality of errors and homogeneity of variance.

## Results

A total of 1,103 groups of cows (*n* = 3,963 cows) were observed. The size of the groups of cows ranged between one and 55, with a general mean of three. The descriptive analysis of the behavioral events observed in the cows in each stage is shown in Table 2. Slips and vocalizations were more often observed during auction, whereas the other behaviors registered were more frequent during unloading and loading; cows turning back were frequently observed during loading.

**Table 2:** Descriptive analysis of behavioral events observed in the cows during unloading, auction and loading at the livestock markets.

**Stage**	**Cows *N***	**Behavioral events (%)**					
		**Slip**	**Fall**	**Balk**	**Turn**	**Jump**	**Vocalization**
Unloading	1,580	13.7	4.1	11.4	0.5	3.0	7.7
Auction	1,608	15.5	1.0	6.3	1.6	0.9	10.0
Loading	775	9.3	3.6	10.3	15.6	1.2	4.0

Regarding negative tactile human-animal interactions, animal handlers were often observed poking and hitting cows with driving devices, with higher frequency during loading; kicking cows was uncommon (Table 3).

**Table 3:** Descriptive analysis of negative tactile interactions observed in the cows during unloading, auction and loading at the livestock markets.

**Stage**	**Cows *N***	**Negative tactile interactions (%)**		
		**Hits**	**Kicks**	**Pokes with the device**
Unloading	1,580	13.2	4.6	16.3
Auction	1,608	22.6	0.0	6.8
Loading	775	53.9	1.3	85.3

Table 4 shows that the highest mean BEI was observed during unloading in winter (1.10), whilst the lowest one was found during auction in summer (0.34). The NTII was highest during loading, in particular in winter and lowest during auction in summer. For all events a minimum value of zero was found, meaning that in some groups no negative welfare events were registered.

**Table 4:** Number of groups of cows observed, minimum, maximum, and mean (*SD*) indexes for behavioral events during movement at each stage of marketing per season.

**Stage**	**Season**	**Groups**	**Behavioral events index (BEI)**	
			**Min-Max**	**Mean (*SD*)**
Unloading	Winter	127	0–11	1.10 (1.87)
	Summer	130	0–18	0.75 (2.11)
Auction	Winter	385	0–9	0.57 (1.19)
	Summer	332	0–5	0.34 (0.63)
Loading	Winter	54	0–5	0.70 (1.09)
	Summer	75	0–10	0.68 (1.45)

Table 5 shows the results of the multinomial regression model used and the factors that had a significant effect on the BEI. The size of the group was significantly associated with the behavioral events, so that increasing by one the number of cows per group reduced the BEI in 0.03. There was an increase of 0.11 in BEI for every extra unit increase in NTII. The model shows that winter season increased BEI by 0.263 compared to summer. The presence of non-slippery floor at unloading and during auction decreased by 1.338 the BEI compared to cows handled on slippery floors. When cows were not mixed with other cattle categories (comingling), the BEI decreased by 0.551 compared with cows that were mixed.

**Table 5:** Parameter estimate, standard error (*SE*), and significance for the models of average behavioral events index during unloading, penning, and loading of cull cows in the market.

**Explanatory variables**	**Parameter estimate**	**S.E**.	***z*-ratio**	***p*-value**
Constant	2.218	0.264	8.390	0.000
Group size	−0.037	0.009	−4.167	0.000
Negative tactile interactions	0.105	0.018	5.793	0.000
**Season**				
Winter	0.263	0.098	2.679	0.007
Summer	Reference			
**Non slippery floor**				
Yes	−1.338	0.262	−5.115	0.000
No	Reference			
**Comingling**				
No	−0.551	0.086	−6.412	0.000
Yes	Reference			

### Health Indicators of the Cows

Of 1,608 cows observed during auction, 49.8% (*n* = 801) had a very lean body condition, 28.3% (*n* = 455) had udder problems, 24% (*n* = 386) were lame, 8.7% (*n* = 140) presented tegumentary lesions, and 3.1% (*n* = 50) had abnormalities of the tail; all health problems were observed in higher percentages in winter than in summer. When exploring the relationship between price per kg live weight (Chilean pesos) and the health variables, the multinomial regression model showed that a very lean body condition, lameness, and presence of tegumentary lesions affected negatively the selling price of the culled cows (Table 6).

**Table 6:** Parameter estimate, standard error (S.E.), and significance for the models of health and price per kg live weight during auction of cows in the market.

**Explanatory variables**	**Parameter estimate**	**S.E**.	***z*-ratio**	***p*-value**
	**Price (Chilean pesos/kg live weight)**			
Constant	828	21.4	38.660	<0.001
Very lean body condition	−84	8.9	−9.475	<0.001
Lameness	−23	10.4	−2.268	0.023
Tegumentary lesion	−77	15.8	−4.877	<0.001

The multinomial regression model indicates that cows with a very lean body condition had a 10% lower final price compared to cows with an appropriate (regular) body condition. The presence of lameness in cows reduced the selling price by 3% compared to not lame cows; the presence of tegumentary lesions reduced the price by 9% compared to cows without lesions. On the other hand, udder problems and tail abnormalities did not significantly affect the price of the cows, and therefore were not included in the model.

## Discussion

Results of the present study are based on the evaluation of behavioral and health indicators related to the welfare of the cows, as observed at the stages of unloading, in the auction ring, and loading at livestock markets. In general, it was found that cows presented a high proportion of negative behavioral events and health problems that reflect a poor welfare status, particularly during winter. The negative tactile interactions by the handlers were directly related to the behavioral events observed in the cows and the poor health status of the cull cows was also related to the price obtained for the cows. The results regarding negative tactile interactions by handlers and infrastructure problems when handling cull cows at livestock markets are similar to findings of earlier studies at this type of premises when observing different cattle categories in general (6, 9, 10) or just weaned calves (11). The welfare of cull cows has been assessed before at slaughterhouse level, at arrival, and during lairage, in Colombia (15, 25) and Chile (18). However, this is the first study trying to assess the welfare of the cows when passing through livestock markets using live animal-based indicators (health and behavior) as outputs and relating these to the handling and some other factors (facilities, comingling).

### Behavioral Events of the Cows

Falls, slips, jumps, balking, turning, and vocalizations during driving of animals are behavioral events associated with fear and discomfort and their measurement can therefore be used to identify a welfare problem (11, 20, 26, 27).

According to Grandin (20) falls of animals during handling are acceptable up to 1%, slips and vocalizations of animals are acceptable up to 3%; an increase in these proportions reflects poor welfare. Our descriptive results for the behavioral events (Table 2) indicate that the presence of slips, falls, and vocalizations during unloading, auction and loading at cattle markets, were higher than recommended and possibly generate a decrease in the welfare of the cull cows during marketing. Our results for cows are in agreement with a study at 18 livestock markets in the United Kingdom, where Gregory et al. (9) also observed that the main welfare problems in fat cattle and calves marketed were slips and falls.

Turns and balks were more frequent during loading of the cows (Table 2). Moreover, negative tactile interactions by the handlers were also high at loading (Table 3). Both the high frequency of cow's turns and backs, and of NTI by handlers could reflect a problem with loading facilities, distractors at loading and also with the lighting and tiredness of the handlers, as it was observed that loading took place at the end of the marketing day and often occurred at night time.

Jumps were uncommon in the cows, whereas this behavior has been frequently observed in weaned calves during marketing (11). The results of the present study showed that some behavioral indicators (falls, slips, jumps, and vocalizations) were higher during unloading compared to loading, which suggests that this stage also generates discomfort and stress in the cows, probably due to the fact that they arrive at a new environment, where they are handled roughly by people. The aforementioned results, together with the health problems observed probably make cull cows more prone to lose balance, slip or fall, and hence decrease their welfare when passing through livestock markets.

### Negative Tactile Interactions With Handlers

NTI like hitting, poking, kicking, and even tail twisting have been often observed during handling at cattle markets as it has been reported in earlier studies in the United Kingdom (4, 9); in Bangladesh (28); in Colombia (29, 30) and also in Chile (8, 10, 11). The most common NTI in the present study were hits and pokes, whereas kicking was uncommon (Table 3). The most common devices used in these interactions during loading and unloading were wooden sticks, sticks made of plastic pipe pieces and electric prods; during auction the most frequently driving device used was a plastic flap. It is worth mentioning that kicking, hitting and poking are banned by the Chilean regulation (23). Our finding is similar to an earlier study in Chile (8) where it was found that forbidden human-animal interactions were still present in 85.7% of the cattle markets evaluated. Considering that the regulations that prohibit these handlings were passed in 2013, more auditing by the competent authority is needed. The Chilean regulation (23) also includes compulsory training of the personnel handling the animals at cattle markets. Although in the view of the authors there have been improvements in several cattle markets compared to de Vries (10) and Sepúlveda (8), more supervision of the behavior of the personnel during the different stages of marketing is required by market managers (usually veterinarians) as well as by the competent authority.

Various authors recommend evaluating handling indicators because these trigger animal behaviors related to fear and escape, like slips, falls, jumps, balking, aggressions, and vocalizations (31–34). These indicators also reflect the efficiency with which animals are handled by the personnel (11, 26, 27, 35). We found that most NTI occurred during loadings, particularly during winter, and the least occurred during auction in summer (Table 4). The loading of animals is the most critical point of those evaluated at the cattle markets, which had also been pointed out by Maria et al. (22). Considering this, the stage of loading animals would be a good one to observe for assessing welfare problems at livestock markets.

There was a significant direct relationship between the BEI and NTII, so that BEI increased when inappropriate handling like kicking, poking, and hitting increased. Our results are in accordance with an earlier study in calves in the UK, where the authors observed that 11% of calves fell during loading when they were inappropriately handled (9) and with a recent study of the behavior of calves at the same markets, in Chile (11). In order to reduce the impact of NTI on BEI and improve the welfare of animals at livestock markets, it is important that stockmen are trained and act according to the existing legislation.

### Health Indicators

Health problems are obvious reasons for culling cows (36) and therefore it was not surprising to find a high proportion of cull cows with this type of problem at livestock markets. A study conducted in Chile determined that the main causes of elimination of dairy cows are reproductive problems, mammary gland affections, and lameness (37). Coincidently, our study showed that a high percentage of the cows arriving at the livestock markets had at least one health problem, mainly very lean body condition (49.8%), udder problems (28.3%), and lameness (24%). These results are also in agreement with studies of cull cows conducted in slaughterhouses in Colombia (25), in Sudan (38), and in Denmark (36). Similar results were obtained in a parallel study of cull cows arriving directly from farms to a slaughterhouse in Chile, where 52% of the cows presented one or more of the same health problems (18).

Considering the chronic characteristics of most of the health problems recorded in the cull cows, the fact that the cows were transported for a short time (~4 h) directly from the farms of origin to the livestock markets and the short time spent in the different stages at the livestock market (~12 h), the high prevalence of health problems most likely indicates that the cows were already sick or in poor body condition when leaving the farms of origin. Notwithstanding this, future research is needed at farm level in order to record the health problems present in cull cows before transport to the livestock markets or slaughterhouses and the cow's fitness for transport, as the clinical condition of them may deteriorate during transport (36). On the farm, cull cows with low body condition are at a greater risk of suffering of other illnesses (39). At slaughter, the low body condition is a risk factor for increasing the number of bruises on the carcass and for carcass condemnations (18), generating economic losses. That is why Grandin (20) and Losada et al. (27) recommend to cull cows before they are in such a bad state, when their welfare and health is severely compromised. Considering all the above mentioned, selling cull cows through cattle markets in these bad health conditions represents an additional effort and is negatively affecting their welfare during transport and marketing in comparison to younger and healthier animals like calves, steers, and heifers.

Culling the cows before they are in poor condition could also have economic benefits, because a higher price could be reached and/or the carcass will not be condemned after slaughter (18). With respect to the sale price in the market, we found a statistical association between the price per kg live weight and the health variables, where the body condition score, presence of lameness, and the tegumentary lesions negatively affected the selling price of the culled cows. Similar results were found in livestock markets in Canada, where cows in poor body condition or abnormal gait were sold at lower prices compared to cows in good body condition (40). From an animal welfare point of view and considering economic losses, it would be better to send culled cows directly from farms to slaughterhouses instead of passing through the cattle markets, particularly those in low body condition and with health problems.

### Other Factors

Several authors mention that there are more slips and falls when the concrete floor is wet or dirty with urine and feces (9, 11, 31), which is in line with the results of the present study (Table 5), where the BEI increased in winter compared to summer, (i.e., when it was rainy). Facilities become slippery when wet and there is probably also an effect of the speed at which the handlers move the animals, trying to finish work quickly with bad weather conditions, as livestock markets in Chile lack roofing. We found that the lack of appropriate floor type was a predictor variable for an increase in the BEI. Often when the floor was described as slippery, we noticed that the surface was slippery due to wear through heavy use over the years. Similar results have been described by Gregory et al. (9); these authors mention that in order to solve this problem, some markets in the United Kingdom have put resin on the concrete floor, particularly on high risk areas, and have observed reductions in slipping. Considering that many cull cows arrive in poor body condition or lame, and have difficulties in moving, it is of primary importance to keep surfaces non-slippery.

Another important factor to be considered when moving the cows at livestock markets is the group size (Table 5); when cattle are separated from their original groups, they are more difficult to be handled (41). In this case we had small groups of cows (mean = 3), ranging between one and 55, and coincidently, we found a significant association between the size of the cow group and the BEI, so that increasing the number of cows in the group reduced the BEI. This result is similar to that found for weaned calves at the same livestock markets (11) and coincides with Grandin (41) that groups of cattle are easier to move than one animal alone. Mixing unfamiliar cattle from different sources or with other categories of cattle has often been observed at Chilean markets (10, 11). Comingling triggers agonistic behaviors related to dominance, like mounting and aggressions; further on this implies movement of animals that can end in cattle slipping, falling, balking, turning, and vocalizing (11, 41). In our study comingling cull cows with other sources and categories of cattle was associated with a higher BEI and hence should be avoided for improving welfare, besides of its effect on the transmission of diseases.

## Conclusions

During marketing of cull cows, negative tactile interactions by handlers, like hitting, poking, and kicking were associated with an increase in behavioral events in the cull cows, like falling, slipping, jumping, turning, and backing. A small group size, winter season, presence of slippery floor, comingling with other cattle categories were also associated with an increase of behavioral events in the cows.

The high proportion of cull cows presenting low body condition and chronic health problems like lameness, udder problems, and others during auction reflect that there is a welfare problem not only at market level, but also on farm. Because sick and low body condition cows have more difficulties when they need to be moved, loaded, and unloaded several times, it is recommended that cull cows should be sold directly from farm to slaughterhouse and considering fitness for transport, in order to avoid further welfare problems and to reduce economic losses from this cattle category.

## Data Availability Statement

The raw data supporting the conclusions of this article will be made available by the authors, without undue reservation.

## Ethics Statement

The animal study was reviewed and approved by Bioethics Committee Use of Animals in Research at Austral University of Chile (Application N325/2018).

## Author Contributions

MS-H and VB were responsible for the elaboration of behavior and handling protocols, general design of the study, and organization of the visits to the livestock markets. MS-H was responsible for the elaboration of health protocols, the collection of behavior, handling, and health data. CG was responsible for contacting livestock managers, general supervision, and acquisition of funding. VB did the statistical analyses and modeling of data. All authors contributed to the writing and discussion of the manuscript and approved its final version.

## Conflict of Interest

The authors declare that the research was conducted in the absence of any commercial or financial relationships that could be construed as a potential conflict of interest.
